# FKBP12 Activates the Cardiac Ryanodine Receptor Ca^2+^-Release Channel and Is Antagonised by FKBP12.6

**DOI:** 10.1371/journal.pone.0031956

**Published:** 2012-02-21

**Authors:** Elena Galfré, Samantha J. Pitt, Elisa Venturi, Mano Sitsapesan, Nathan R. Zaccai, Krasimira Tsaneva-Atanasova, Stephen O'Neill, Rebecca Sitsapesan

**Affiliations:** 1 School of Physiology & Pharmacology, Centre for Nanoscience and Quantum Information, and Bristol Heart Institute, University of Bristol, Bristol, United Kingdom; 2 Department of Engineering Mathematics, University of Bristol, Bristol, United Kingdom; 3 Cardiovascular Research Group, Core Technology Facility, University of Manchester, Manchester, United Kingdom; University of Queensland, Australia

## Abstract

Changes in FKBP12.6 binding to cardiac ryanodine receptors (RyR2) are implicated in mediating disturbances in Ca^2+^-homeostasis in heart failure but there is controversy over the functional effects of FKBP12.6 on RyR2 channel gating. We have therefore investigated the effects of FKBP12.6 and another structurally similar molecule, FKBP12, which is far more abundant in heart, on the gating of single sheep RyR2 channels incorporated into planar phospholipid bilayers and on spontaneous waves of Ca^2+^-induced Ca^2+^-release in rat isolated permeabilised cardiac cells. We demonstrate that FKBP12 is a high affinity activator of RyR2, sensitising the channel to cytosolic Ca^2+^, whereas FKBP12.6 has very low efficacy, but can antagonise the effects of FKBP12. Mathematical modelling of the data shows the importance of the relative concentrations of FKBP12 and FKBP12.6 in determining RyR2 activity. Consistent with the single-channel results, physiological concentrations of FKBP12 (3 µM) increased Ca^2+^-wave frequency and decreased the SR Ca^2+^-content in cardiac cells. FKBP12.6, itself, had no effect on wave frequency but antagonised the effects of FKBP12.

We provide a biophysical analysis of the mechanisms by which FK-binding proteins can regulate RyR2 single-channel gating. Our data indicate that FKBP12, in addition to FKBP12.6, may be important in regulating RyR2 function in the heart. In heart failure, it is possible that an alteration in the dual regulation of RyR2 by FKBP12 and FKBP12.6 may occur. This could contribute towards a higher RyR2 open probability, ‘leaky’ RyR2 channels and Ca^2+^-dependent arrhythmias.

## Introduction

The cardiac ryanodine receptor (RyR2) is the main pathway for the release of intracellular Ca^2+^ during excitation-contraction (EC) coupling in cardiac muscle [1]. Several studies have shown that FKBP12.6, a member of the FK506-binding protein family, binds with high affinity to RyR2 [2]–[4] but the functional consequences of this interaction has remained a highly controversial subject. The dissociation of FKBP12.6 from RyR2 has been linked with heart failure and arrhythmia generation [5], [6] and it has been proposed that the ensuing dysfunctional RyR2 channel behaviour contributes to the defective Ca^2+^ homeostasis that is characteristic of heart failure [7]. A maximum of four FKBP12.6 molecules is thought to bind each RyR2 tetramer [2]. The dissociation of FKBP12.6 from RyR2 has been reported to induce marked changes to RyR2 function which include pronounced sub-conductance state gating, high open probability (Po) and channel gating that is unregulated by Ca^2+^ [5], [8], [9]. On the basis of this work, FKBP12.6 has become widely accepted as a ‘stabiliser’ of RyR2 channel function but there is an underlying impression that this is an over-simplification of the role of FKBP12.6 as some investigators find that FKBP12.6 appears not to influence RyR2 gating [10]–[12].

Cellular studies are more unanimous in pointing towards a cardioprotective role for FKBP12.6. Many studies indicate that FKBP12.6 does ‘stabilise’ or reduce sarcoplasmic reticulum (SR) Ca^2+^-release [5], [13]–[16], and it appears to provide a protective role that becomes altered in heart failure [7], [14]. Cardiac cells derived from FKBP12.6 knockout mice show altered Ca^2+^-spark characteristics when compared to wild type cells with the amplitude, size and duration of sparks being significantly increased and the gain of Ca^2+^-induced Ca^2+^-release elevated [17].

In contrast to FKBP12.6, there has been less emphasis on the cardiac role of FKBP12. However, the FKBP12 knockout mouse is characterised by severe dilated cardiomyopathy and the RyR2 channels isolated from this model exhibit unusual gating behaviour governed by long-lived sub-conductance state openings [18]. Moreover, Seidler et al. (2007) demonstrate that GST-FKBP12 binds tightly to RyR2 and that overexpression of FKBP12 causes alterations to the characteristics of Ca^2+^-sparks. These results suggested to us that FKBP12 might have a more important cardiac role than previously envisaged, and that a primary target of its action may be RyR2. It is especially interesting therefore, that FKBP12 is thought to be present at much higher concentrations than FKBP12.6 in cardiac cells [2], [19]. FKBP12 shares 85% sequence homology with FKBP12.6 and crystallographic studies show very high structural homology [20]–[22] highlighting the possibility that FKBP12 and FKBP12.6 could compete for the same binding sites on RyR2. We have therefore investigated the ability of FKBP12 and FKBP12.6 to modulate the single-channel function of RyR2 and affect waves of spontaneous Ca^2+^-induced Ca^2+^-release (CICR) in isolated cardiac cells. We demonstrate the novel ability of FKBP12 to activate RyR2. Importantly, FKBP12.6 can antagonise activation of RyR2 by FKBP12 and our data suggest that FKBP12.6 may be a partial agonist with negligible efficacy at RyR2. Our results suggest that FKBP12 and FKBP12.6 can regulate the gating of RyR2 by modulating the sensitivity of the channel to cytosolic Ca^2+^.

## Methods

### Isolation of membrane fractions and planar phospholipid bilayer techniques

Mixed membrane (MM) vesicles were prepared from sheep hearts (obtained from a local abbatoir), as described previously [23]. In brief, homogenised ventricular tissue was subjected to centrifugation at 6500× g followed by ultracentrifugation of the supernatant at 100,000× g. Following this spin, both the sedimented MM pellet and the supernatant (S2) were retained for detection of FKBPs. The MM pellet was resuspended with a solution containing 0.4 M KCl to remove remaining FKBPs and the heavy SR membrane fraction was obtained from a discontinuous sucrose-density gradient, snap frozen in liquid N_2_, and stored at −80°C as previously described [23]. SR vesicles were fused with planar phosphatidylethanolamine lipid bilayers as described previously [23]. The SR vesicles fused in a fixed orientation such that the *cis*-chamber corresponded to the cytosolic space and the *trans*-chamber to the SR lumen. The *trans*-chamber was held at ground and the *cis*-chamber held at potentials relative to ground. After fusion, the *cis*-chamber was perfused with 250 mM HEPES, 80 mM Tris, 10 µM free Ca^2+^, pH 7.2, unless stated otherwise. The *trans*-chamber was perfused with 250 mM glutamic acid and 10 mM HEPES, pH to 7.2 with Ca(OH)_2_ (free [Ca^2+^], approximately 50 mM). Experiments were carried out at room temperature (22±2°C). The identity of the channels was confirmed by the single-channel conductance and the application of caffeine at the end of the experiment. The free [Ca^2+^] and pH of the solutions were determined using a Ca^2+^ electrode (Orion 93-20) and a Ross-type pH electrode (Orion 81-55) as previously described [23]. After addition of 10 mM EGTA to the *cis*-chamber, the approximate free [Ca^2+^] was calculated using the program MaxChelator (www.stanford.edu/~cpatton/maxc.html) as 1 nM. The pH of the solutions was maintained constant. Additions of FKBP12 and FKBP12.6 were made to the *cis*-chamber. Both proteins were stored in a buffer containing 10 mM HEPES/50 mM NaCl, 0.5 mM DTT and volumes added to the *cis*-chamber were <2% of the total volume. Control buffer solution did not alter Po.

### Data acquisition and Analysis

Channel recordings were displayed on an oscilloscope and recorded on digital audiotape (DAT). Current recordings were filtered at 800 Hz (−3 dB) and digitised at 20 kHz using Pulse (HEKA, Elektronik Lambrecht/Pfalz, Germany). Channel open probability (Po) was determined over ≥3 min of continuous recording, unless otherwise stated, using the method of 50% threshold analysis [24]. Lifetime analysis was carried out only when a single channel incorporated into the bilayer. Events <1 ms in duration were not fully resolved and were excluded from lifetime analysis. Individual lifetimes were fitted to a probability density function (pdf) by the method of maximum likelihood according to the equation:
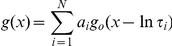
where lnτ*_I_* is the logarithm of the *i*th time constant and *a_i_* is the fraction of the total events represented by that component [25].

### Cardiac myocyte isolation and measurement of Ca^2+^ waves in rat permeabilised myocytes

Male Wistar rats were killed by cervical dislocation in accordance with the Animal (Scientific Procedures) Act 1986. Rat ventricular myocytes were isolated using a protease and collagenase Langendorff perfusion technique as previously described [26]. Cells were incubated with 0.1 mgml^−1^ β-escin for 1 min to permeabilise the surface membrane as previously described [27]. The β-escin was removed by hand-centrifugation. After permeabilisation myocytes were resuspended in a mock intracellular solution composed of (mM): 100 KCl, 5 Na_2_ATP, 5.4 MgCl_2_, 25 Hepes, 0.1 K_2_EGTA, pH 7.0 at 37°C for 5 min. This was to encourage dissociation of endogenous FK-binding proteins from RyR2 as it has been shown that FK-binding proteins dissociate quickly at 37°C [2]. Spontaneous waves of CICR were measured by adding 10 µM Fluo-5F to the mock intracellular solution and raising the [Ca^2+^] to 234 nM. The waves were recorded at 21°C using a laser-scanning confocal microscope (BioRad MRC 1024) in the linescan mode. Linescan images were analyzed using ImageJ software (wsr@nih.gov). Waves of CICR were thus monitored in each cell before and after the addition of either 3 µM FKBP12 or 200 nM FKBP12.6 to the mock intracellular solution. For experiments to test the ability of FKBP12.6 to antagonize FKBP12, cells were first incubated with 200 nM FKBP12.6 for 5 min at 21°C and subsequently perfused with 3 µM FKBP12 in the continued presence of FKBP12.6. The SR Ca^2+^-content was examined in the absence and in the presence of FKBP12 after application of caffeine (20 mM) to release Ca^2+^ stores into the cytosol.

### Cloning, expression and purification of FK-506 binding proteins

Recombinant DNA techniques were carried out according to standard protocols. The FKBP12 and 12.6 proteins were constructed by PCR amplification using the pCMV6-XL4-true clone (OriGene Technologies, Rockville, USA) and the PGEX-4T-1 vector (Addgene, Cambridge, USA) respectively. The two constructs were subcloned in the pET-28a plasmid (Novagen, USA) and transformed in *Escherichia coli* BL21(DE3) Codon Plus cells (Stratagene, CA, USA). Protein expression was induced by addition of Isopropyl-β-D-1-thiogalactopyranoside (IPTG 1 mM) to the culture. After 3 hours, cells were pelleted, resuspended in Tris-buffer (10 mM Tris-HCl, 5 mM β-mercaptoethanol, 1 mM EDTA), and lysed by sonication to obtain soluble protein. The protein extracts were purified by cation exchange and size exclusion chromatography using an HPLC-AKTA machine (GE healthcare, Buckinghamshire, UK). The filtered proteins were loaded in a CM Sepharose Fast Flow column (GE healthcare, Buckinghamshire, UK) in Tris-buffer, pH 6.8 and pH 6.6 respectively for FKBP12.6 and FKBP12, and eluted by a linear NaCl gradient. The proteins were subsequently purified by gel filtration using a Superdex 75 10/300 GL column (GE healthcare, Buckinghamshire, UK) in a buffer containing 10 mM HEPES, 50 mM NaCl, 0.5 mM DTT, pH 8. The purified chromatographic peaks were checked by SDS-polyacrylamide gel electrophoresis. The high-quality samples were mixed and concentrated with Amicon 10 kDa Ultra-4 Centrifugal Filter Units (Millipore, Watford, UK) by centrifugation at 4°C. The final protein concentration of FKBP12 and FKBP12.6 was calculated by a Bradford assay [28] and both the quality and the identity of the proteins were validated by Mass Spectrometry and N-terminal sequencing. FKBP12 was also purchased from Sigma-Aldrich (Dorset, UK).

### Western Blotting

The protein concentration of the vesicles was calculated by a Bradford assay. Proteins were then size fractionated by SDS-PAGE on either a 4–20% precast gel (BioRad, Hertfordshire, UK) or a 15% gel. Following separation, proteins were transferred to a nitrocellulose membrane and non-specific binding sites were blocked for 1 hr at room temperature using 5% dried milk and Tris-buffered saline, 0.1% Tween 20, pH 7.4. Membranes were probed for 2 h at room temperature with a polyclonal primary anti-FKBP antibody ((1∶500) Thermo Fisher Scientific, Leicestershire, UK)). A secondary horseradish peroxidase linked anti-rabbit IgG ((1∶5000) GE Healthcare, Buckinghamshire, UK)) was used in combination with an enhanced chemiluminescent detection system (GE Healthcare, Buckinghamshire, UK) to visualise the primary antibodies.

### Detection and identification of FKBP12 and FKBP12.6 in sheep heart

During the procedure for isolation of heavy SR membrane vesicles from sheep cardiac muscle, the MM vesicle fraction, the corresponding supernatant (termed S2; see above) and the heavy SR vesicles were collected and electrophoresed on a 15% SDS-PAGE. Prior to electrophoresis, the supernatant (S2) was filtered multiple times, each time reducing the pore of the filter from 1.2 µM–0.22 µM and concentrated by centrifugation at 3500× g for 15 h with an Amicon 10 kDa Ultra-15 centrifugal filter unit (Millipore, Watford, UK). Following separation, the proteins were either visualised by staining with Coomassie brilliant blue (CBB) or transferred to a nitrocellulose membrane for Western blot analysis. Where anti-FKBP antibodies indicated the presence of FKBPs on the Western blot, the corresponding band visualised by CBB staining was cut from the gel. The gel slice was subjected to in-gel tryptic digestion using a ProGest automated digestion unit (Digilab UK). The resulting peptides were fractionated using a Dionex Ultimate 3000 nanoHPLC system. In brief, peptides in 1% (v/v) formic acid were injected onto an Acclaim PepMap C18 nano-trap column (Dionex-Thermo Fisher Scientific, Leicestershire, UK). After washing with 0.5% (v/v) acetonitrile 0.1% (v/v) formic acid peptides were resolved on a 250 mm×75 µm Acclaim PepMap C18 reverse phase analytical column (Dionex-Thermo Fisher Scientific, Leicestershire, UK) over a 120 min organic gradient with a flow rate of 300 nlmin^−1^. Peptides were ionized by nano-electrospray ionization at 2.3 kV using a stainless steel emitter with an internal diameter of 30 µm (Proxeon-Thermo Fisher Scientific, Leicestershire, UK). Tandem mass spectrometry analysis was carried out on a LTQ-Orbitrap Velos mass spectrometer (Thermo Fisher Scientific, Leicestershire, UK). The Orbitrap was set to analyze the survey scans at 60,000 resolution and the top twenty ions in each duty cycle selected for MS/MS in the LTQ linear ion trap. Data was acquired using the Xcalibar v2.1 software (Thermo Fisher Scientific, Leicestershire, UK). The raw data files were processed and quantified using Proteome Discoverer software v1.2 (Thermo Fisher Scientific, Leicestershire, UK) with searches performed against the UniProt human database by using the SEQUEST algorithm with the following criteria; peptide tolerance = 10 ppm, trypsin as the enzyme, carboxyamidomethylation of cysteine as a fixed modification and the oxidation of methionine as a variable modification. The reverse database search option was enabled and all data was filtered to satisfy false discovery rate (FDR) of less than 5%.

### Computations

Monte Carlo simulations of the two-state RyR ion channel model were performed using XPPAUT (www.math.pitt.edu/~bard/xpp/xpp.html). Steady state Po was computed by taking the trial average of 100 simulations of one channel. For plotting the modelling results we used IGOR Pro (http://www.wavemetrics.com/).

### Statistics

Data are expressed as mean ± SEM where n≥4. For n = 3, SD is given. Where appropriate, Student's *t*-test was used to assess the difference between treatments. Where multiple treatments were compared, ANOVA followed by a modified t-test was used to assess the difference between treatments. A *p* value of <0.05 was taken as significant.

### Materials

Escin was obtained from Sigma-Aldrich (Dorset, UK). Fluo-5F was obtained from Invitrogen, (Paisley, UK). Other chemicals were AnalaR or the best equivalent grade from BDH (Poole, UK) or Sigma-Aldrich (Dorset, UK). All solutions were made in deionised water and those for use in bilayer experiments were filtered through a Millipore membrane filter (0.45 µm pore).

## Results

### Effects of FK-binding proteins on RyR2 single channel function

**Figure 1:**
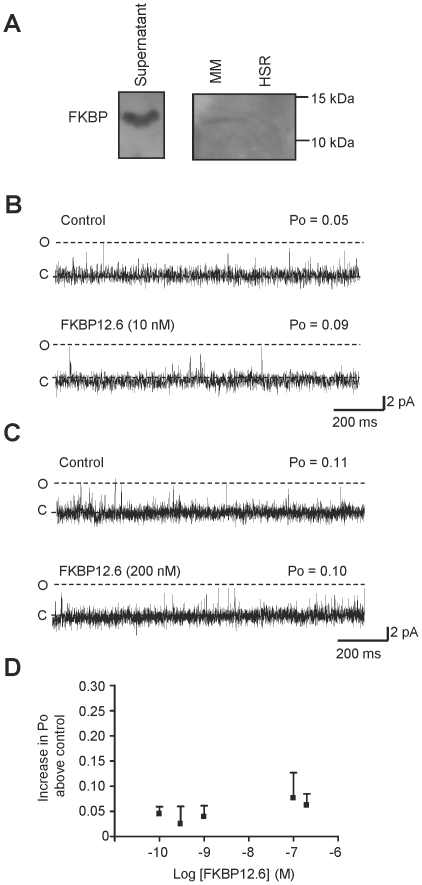
The effects of FKBP12.6 on RyR2 gating. (A) Western blot analysis of the supernatant (left gel) and sedimented cardiac mixed membrane vesicles (left lane of right gel) and heavy SR vesicles (right lane of right gel). Vesicles were loaded onto 15% gels at 1 mg. Western blots were probed with an anti-FKBP12 antibody (recognising both FKBP12 and FKBP12.6). Size markers are indicated in kDa. Two representative single-channel experiments are shown. One experiment illustrates the typical effects of 10 nM FKBP12.6 (B) and the other illustrates the effect of a concentration of FKBP12.6 (200 nM) that is expected to be higher than the physiological level (C). The dotted lines indicate the open (O) and closed (C) channel levels respectively. (D) The relationship between FKBP12.6 concentration and Po is shown. The Po is expressed as the increase in Po above the control level (for each channel, the control Po was subtracted from the Po in the presence of FKBP12.6). Error bars show the mean values ±SEM (n≥4).

The endogenous FKBPs associated with RyR2 were dissociated by high salt incubation during the sucrose gradient step of the heavy SR isolation procedure (see Figure S1). For single-channel studies this treatment is preferable to treatment with drugs such as rapamycin or FK-506 as we found, in line with other investigators [29], [30], that these compounds produced direct and irreversible effects on RyR2 channel gating (results not shown) that were not related to the dissociation of FKBPs. Figure 1A demonstrates that FKBPs are not detected in the heavy SR membrane fraction but also illustrates that most of the FKBPs are not associated with RyR2 but are present in the supernatant of the second spin (which is normally discarded). To determine the identity of the FKBPs present in sheep cardiac muscle, where FKBPs were detected by Western blot analysis, we subjected the same samples (supernatant of the second spin (S2) and the MM vesicles) to electrophoresis using an SDS-polyacrylamide gel under identical conditions to the Western blot. Following CBB staining, these bands were cut out and sequenced using mass spectrometry as described in the Methods. In both the supernatant of the second spin (S2) and in the MM vesicles, FKBP12 was detected with high confidence (false discovery rate <1%). FKBP12.6 was detected in both fractions with lower confidence (>5%).

The FKBP-depleted SR was then incorporated into bilayers and two typical experiments are shown, illustrating that neither a low (Figure 1B) nor a high (Figure 1C) concentration of FKBP12.6 produced any decrease in Po and that no large changes in gating behaviour could be detected. Notice that there are no obvious sub-conductance gating states (before or after addition of FKBP12.6), although filtering the data causes truncation of the very brief events. The physiological levels of FKBP12.6 are not known but are thought to be very low in cardiac cells [2], [3], [10]. Over a wide range of concentrations, likely to encompass the physiological levels, FKBP12.6 caused what looked like a very slight increase in Po although this was only significant at 200 nM (Po increased from 0.06±0.01 to 0.12±0.02 (SEM; n = 12; *p*<0.05)). RyR channels are known to exhibit a large variation in their Po levels [31]–[34] and therefore to investigate if variation in the control Po could mask any small effects of FKBP12.6, we subtracted the control Po of each channel from the Po obtained in the presence of FKBP12.6. Figure 1D illustrates this analysis showing that if the variability in control Po is corrected for, there appears to be a trend towards a very slight increase in Po.

**Figure 2:**
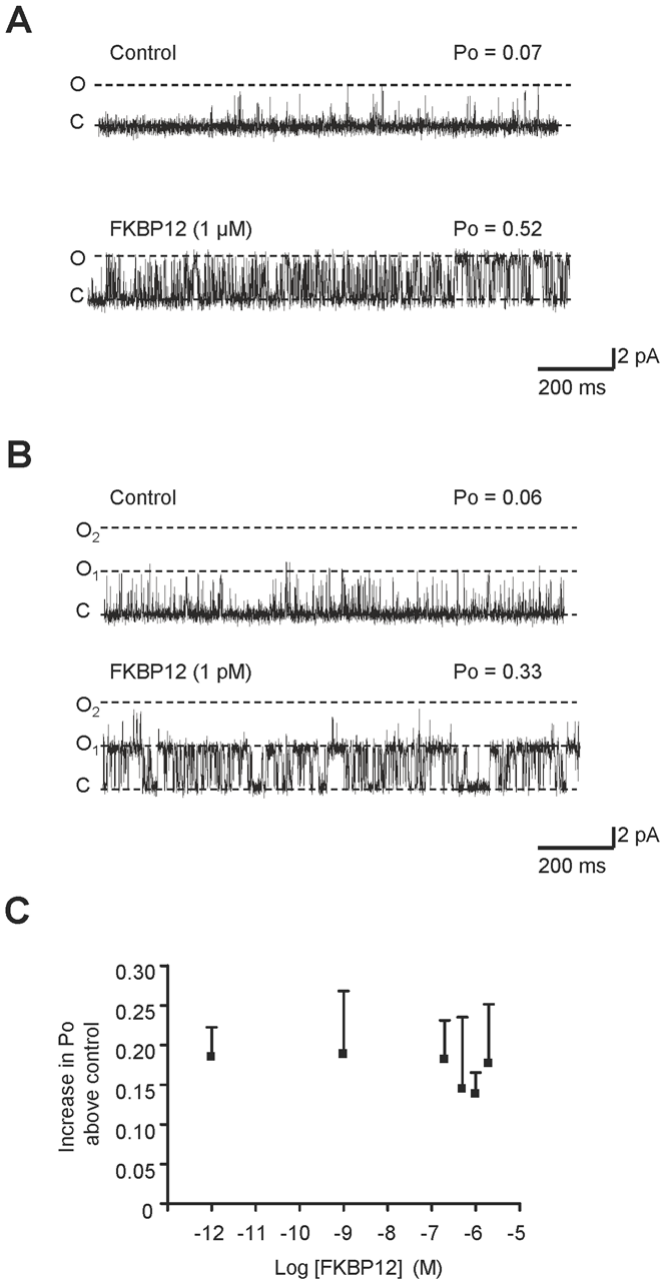
The effect of FKBP12 on RyR2 gating. Two typical single-channel experiments demonstrating that addition of either 1 µM (A) or 1 pM (B) cytosolic FKBP12 causes a marked increase in channel Po. The dotted lines indicate the open (O, O_1_, O_2_) and closed (C) channel levels. (C) The relationship between FKBP12 concentration and Po is shown. Po is expressed as the increase in Po above the control level (for each channel, the control Po was subtracted from the Po in the presence of FKBP12). Error bars show the mean values ±SEM (n = 4–17).

Although cardiac FKBP12.6 levels are extremely low, FKBP12 is present at much higher concentrations (1–3 µM) [3], [10]. Figure 2A shows a typical experiment illustrating that a physiological concentration of FKBP12 is very effective as an activator of RyR2. We found that even very low concentrations of FKBP12 (1 pM) could activate RyR2 (see Figure 2B) indicating that FKBP12 has high affinity for RyR2. 1 pM FKBP12 increased Po from 0.04±0.008 to 0.23±0.04 (SEM; n = 6; **p<0.01). FKBP12 was not able to fully open the channel at any concentration (Figure 2C) indicating that FKBP12 is a partial agonist of RyR2 but with much greater efficacy than FKBP12.6. As with the FKBP12.6 data (Figure 2C), to remove any possible dependence on the starting Po level, we subtracted the control Po of each channel so that only the increase in Po is compared for each concentration of FKBP12.

**Figure 3:**
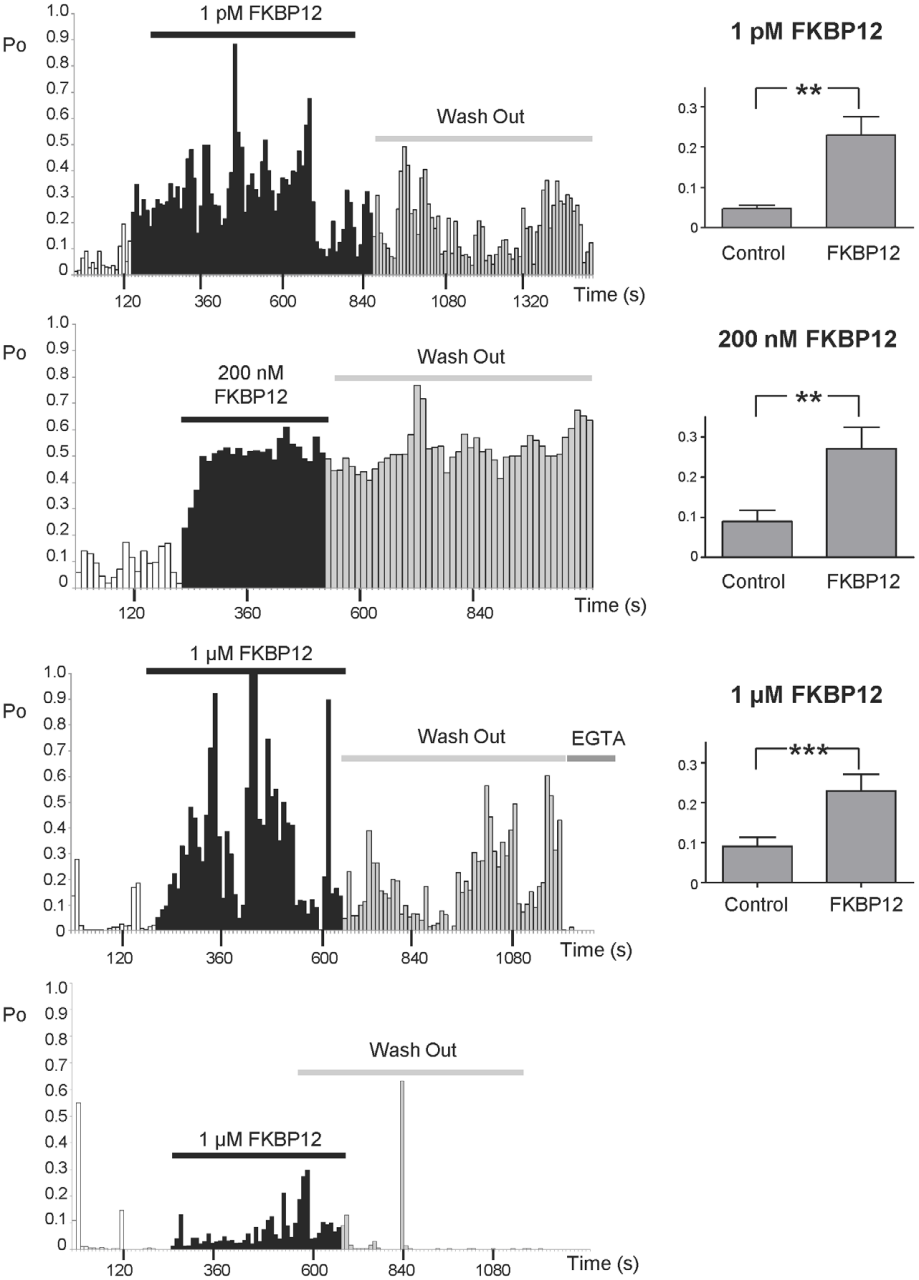
Time dependence of the effects of FKBP12 and washout of FKBP12. The data from four different experiments are shown to illustrate the wide variation in RyR2 Po that occurs over time, even during the control period (before addition of FKBP12). Such shifts in Po (modal gating) are characteristic of RyR2 channel gating [32], [35]. Single-channel records were divided into 10 s segments and the Po of each segment was calculated. Po was then plotted against time for each experiment. Before addition of FKBP12, channels were activated solely by 10 µM cytosolic Ca^2+^. Typical examples of the effects of 1 pM, 200 nM and 1 µM FKBP12 in the continued presence of 10 µM cytosolic Ca^2+^ are shown. The time of incubation with the indicated FKBP concentration is shown by the bars, as is the time following perfusion of the cytosolic chamber back to control solutions in order to washout the added FKBP12. The mean data obtained from the steady-state Po changes (recorded for ≥3 min of continuous recording) resulting from the addition of FKBP12 at the indicated concentrations are shown in the bar charts to the right of the example Po-time plots. Po was 0.04±0.008 and 0.23±0.04 (SEM; n = 6; **p<0.01) before and after 1 pM FKBP12, 0.08±0.03 and 0.27±0.05 (SEM; n = 17; **p<0.01) before and after 200 nM FKBP12 and 0.09±0.23 and 0.23±0.04 (SEM; n = 28; ***p<0.001) before and after 1 µM FKBP12.

The fact that a wide range of FKBP12 concentrations produce similar increases in Po indicates that when a molecule/s of FKBP12 binds to RyR2, it binds very tightly and dissociates slowly. If this is the case, we would expect Po to remain high after washout of FKBP12 from the cytosolic chamber. Where possible (because bilayers often break with perfusion), we therefore recorded for long periods (9 min) after washing out the FKBP12 in order to investigate if dissociation of FKBP12 from RyR2 could be detected by a sudden drop in Po. Figure 3 shows examples of the time-dependence of Po that was observed after incubating RyR2 in the presence of low (1 pM), medium (200 nM) or high (1 µM) concentrations of FKBP12. Po was measured in 10 s segments. The mean steady-state Po change for the indicated FKBP12 concentration is shown in the bar charts. RyR2 is known to exhibit modal gating [32], [35] and this was very obvious both in the control records and after addition of FKBP12. Modal gating was also evident following washout of FKBP12 but in the majority of experiments, there were no obvious changes in Po that could signal the sudden dissociation of FKBP12 from RyR2 (see first three examples of Po time plots). Sometimes after washout, the average Po was slightly lower than in the presence of FKBP12 (see plots 1 and 3) but was still higher than the control (before addition of FKBP12). In only 3 out of 17 washout experiments did Po appear to completely reverse back to control values following FKBP12 washout (see example in bottom panel). These variable changes in Po following FKBP12 washout suggest that dissociation of FKBP12 from RyR2 is a slow process (of the order of minutes) that is difficult to observe within the timescale of a single-channel experiment.

**Figure 4:**
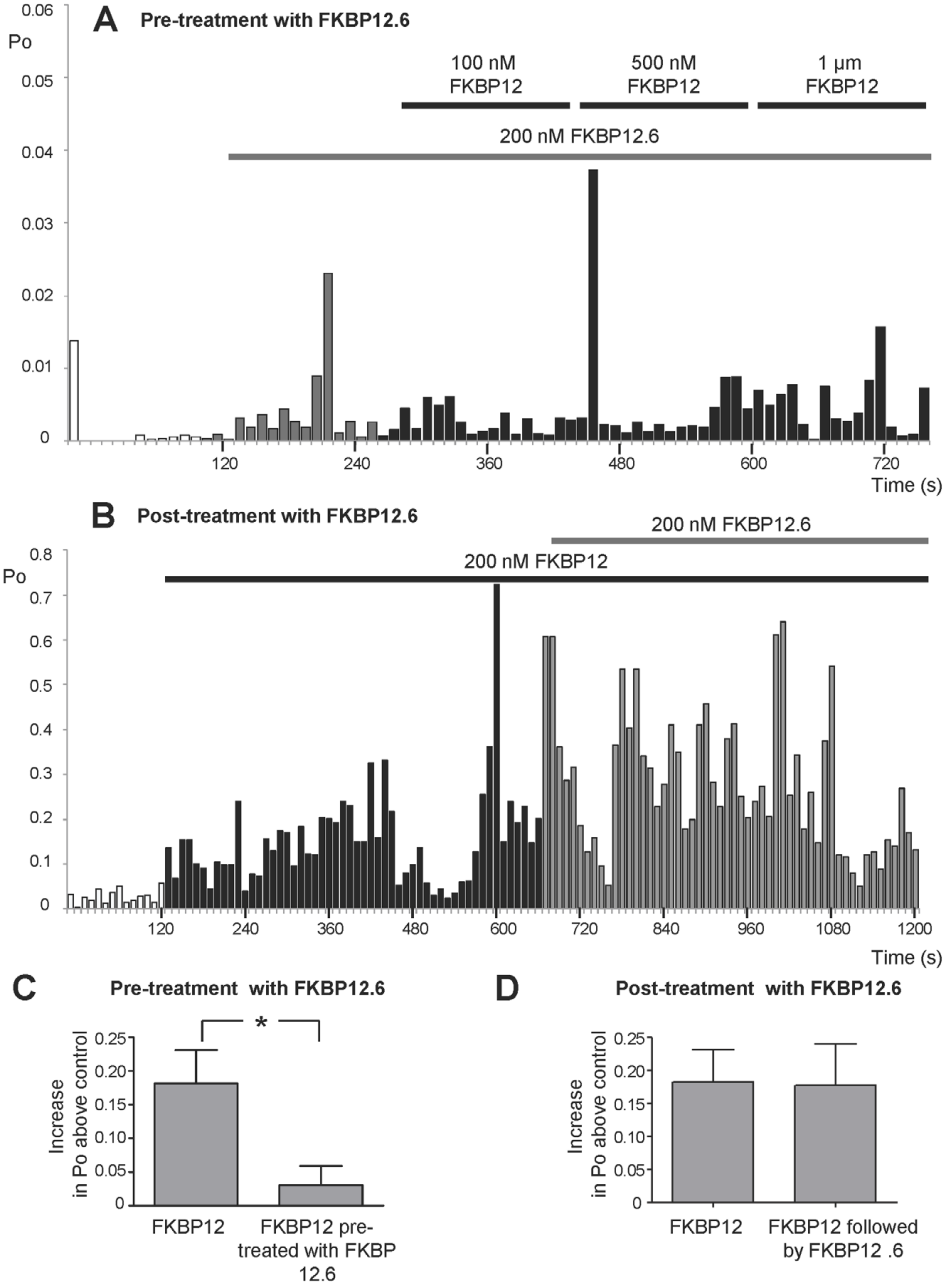
The effects of FKBP12.6 on the ability of FKBP12 to activate RyR2. (A) and (B) are typical examples of experiments where RyR2 Po was calculated in 10 s segments and plotted against time. (A) Pre-treatment with FKBP12.6 (200 nM). Control recordings were obtained in the presence of 10 µM cytosolic Ca^2+^ as the sole channel activator. As indicated by the bar, 200 nM FKBP12.6 was added to the cytosolic chamber. There was no obvious change in Po (perhaps a slight increase occurred but notice that Po, on average, remains well below 0.01) following the addition of 200 nM FKBP12.6. The subsequent sequential additions of increasing levels of FKBP12 did not elicit any obvious increases in Po. (B) Post FKBP12-treatment with FKBP12.6 (200 nM). Control recordings were obtained in the presence of 10 µM cytosolic Ca^2+^ as the sole channel activator. The bar indicates the time of incubation of the channel with 200 nM FKBP12 in the cytosolic chamber. The subsequent addition of 200 nM cytosolic FKBP12.6 (shown by the bar) did not reverse the activating effects of FKBP12. (C) Mean data illustrating that pre-treatment with 200 nM FKBP12.6 (as in the example shown in (A)), prevents channel activation by FKBP12 (1 µM). (D) Mean data showing that, on average, FKBP12.6 (200 nM) does not reverse the activation caused by FKBP12 on the timescale of a single-channel experiment, even when Po is monitored for 9 min after adding the FKBP12.6. Mean values ± SEM (n≥4; *p<0.05) are shown.

Since FKBP12.6 appears to have very little ability to activate RyR2 and since FKBP12 and FKBP12.6 are extremely similar structurally, it is possible that FKBP12.6 could act as an antagonist of FKBP12. Cryo-electron microscopy and 3D reconstruction of RyR1 indicates that FKBP12 and FKBP12.6 bind within the same location on the cytoplasmic face of RyR1 [36]–[38] providing evidence that FKBP12 and FKBP12.6 could compete for the same binding site/s on RyR channels. If this is the case, then the binding of FKBP12.6 to RyR2 should reduce the effectiveness of FKBP12 as an activator of RyR2. Figure 4A shows the time dependence of RyR2 Po in a typical experiment where FKBP12.6 (200 nM) was first added to the cytosolic channel side and this was followed by incremental additions of FKBP12. In the presence of FKBP12.6, FKBP12 was not able to activate the channel to such a high Po as in the absence of FKBP12.6 (notice that the Po axis is highly expanded so that the very low Po levels can be tracked). The mean data in Figure 4C demonstrates that preincubation of RyR2 with FKBP12.6 significantly reduces the ability of FKBP12 to activate RyR2 as expected if FKBP12.6 is acting as an antagonist of FKBP12.

Our washout data (Figure 3) suggests that once bound, FKBP12 dissociates slowly from RyR2. It would be expected therefore, that if FKBP12 and FKBP12.6 compete for the same sites, FKBP12.6 would be unable to access the binding sites if the channels were first pre-bound with FKBP12. We therefore performed experiments where FKBP12 was added before adding FKBP12.6. The results of a typical experiment are shown in Figure 4B and the mean Po levels are given in Figure 4D. In line with our hypothesis, the addition of FKBP12.6 did not immediately lower RyR2 Po.

**Figure 5:**
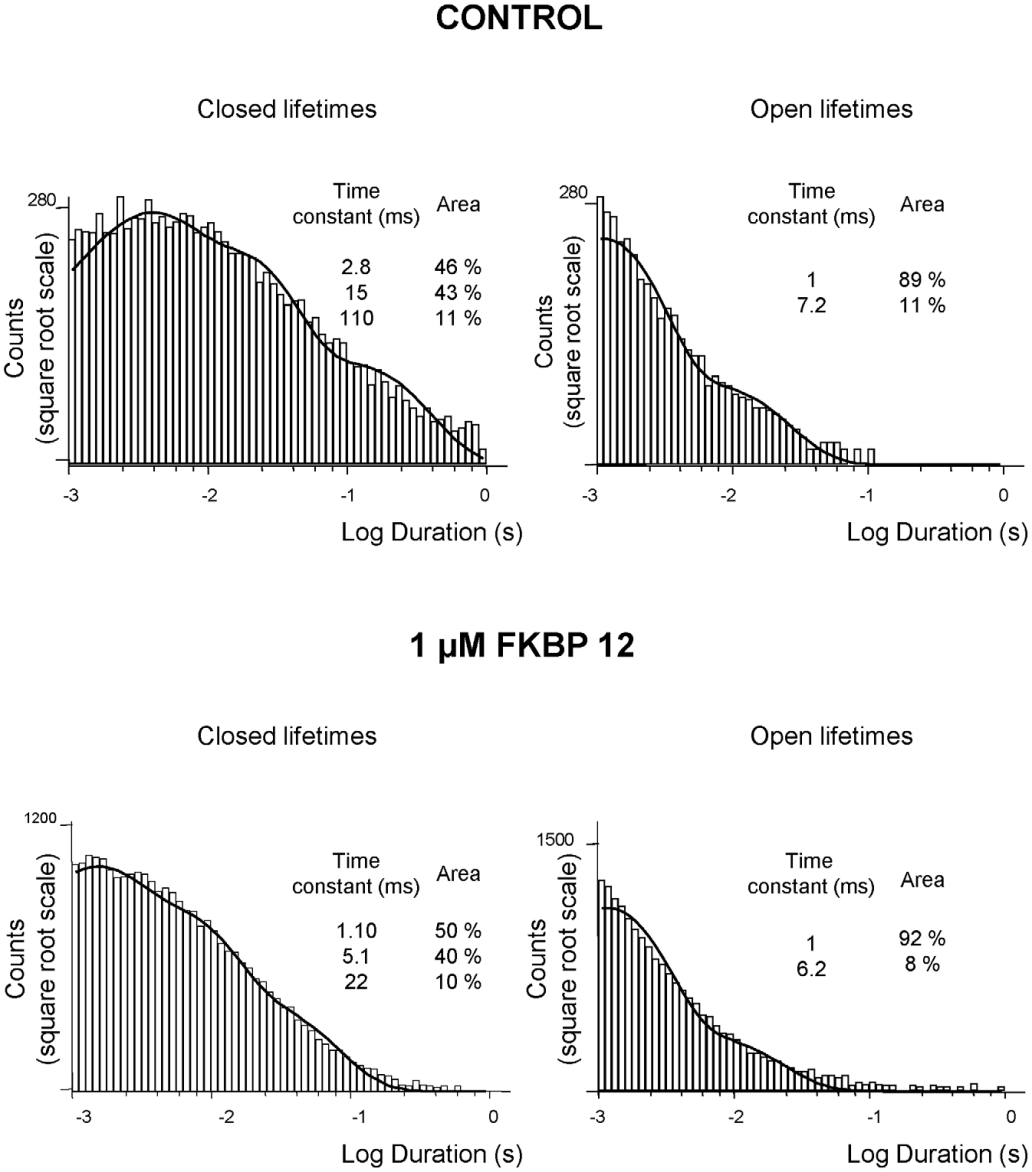
The effects of FKBP12 on open and closed lifetime distributions. The open (right) and closed (left) lifetime distributions and pdfs from a typical single-channel activated by 10 µM Ca^2+^ alone (top panel) and from a channel activated by 1 µM cytosolic FKBP12 (bottom panel) are shown. The best fits to the data were obtained using maximum likelihood fitting. The resulting time constants and percentage areas are shown. Similar results were obtained in 5 further channels.

To investigate the mechanisms underlying RyR2 activation by FKBP12 we performed lifetime analysis on experiments where only a single channel was gating in the bilayer. [In most experiments, multiple channels incorporated in the bilayer and these experiments were used for Po measurements but lifetime analysis could not be performed]. The mean open time was 1.01±0.04 ms in the absence and 1.82±0.24 ms (n = 6; SEM) in the presence of 1 µM FKBP12 and the mean closed time was 27.94±9.98 ms in the absence and 6.93±1.32 ms (n = 6; SEM) in the presence of 1 µM FKBP12. Thus FKBP12 primarily affected the duration of the closed times. This mechanism was confirmed by lifetime analysis and examples of representative open and closed lifetime histograms for control and FKBP12 treated channels are shown in Figure 5. The best fit to control data was obtained with three exponentials to the closed lifetime distribution and two exponentials to the open lifetime distribution indicating at least three closed states and two open states [39], [40]. FKBP12 reduced the duration of all the closed states and increased the proportion of events occurring to the shortest time constant whereas little change in duration of the openings could be detected. Thus, FKBP12 primarily increases Po by increasing the frequency of channel openings. We have previously demonstrated that this is also the mechanism by which cytosolic Ca^2+^ activates RyR2 and the mechanism by which agents such as caffeine that “sensitise” RyR2 to the effects of cytosolic Ca^2+^ appear to increase Po [41]. Hence, it is likely that FKBP12 is increasing Po by sensitising the channel to cytosolic Ca^2+^.

If this is the mechanism by which FKBP12 activates RyR2, then FKBP12 will not activate RyR2 in the absence of activating levels of cytosolic Ca^2+^. We found that this was indeed the case. If the cytosolic [Ca^2+^] was lowered to approximately 1 nM by addition of 10 mM EGTA, then Po was reduced to zero (Po = 0; n = 4). After addition of 1 µM FKBP12, Po remained at zero (n = 4). Similarly, if cytosolic Ca^2+^ was reduced to approximately 1 nM following channel activation by FKBP12 in the presence of micromolar cytosolic Ca^2+^, the channels could be completely shut down. An example of this behaviour can be seen in Figure 3 (third Po time-plot; indicated by ‘EGTA’) where a channel that has been activated by FKBP12 can be closed by lowering cytosolic Ca^2+^ to sub-activating (approximately 1 nM) levels.

### Modelling the effects of FK-binding proteins on RyR2 channel gating

**Figure 6:**
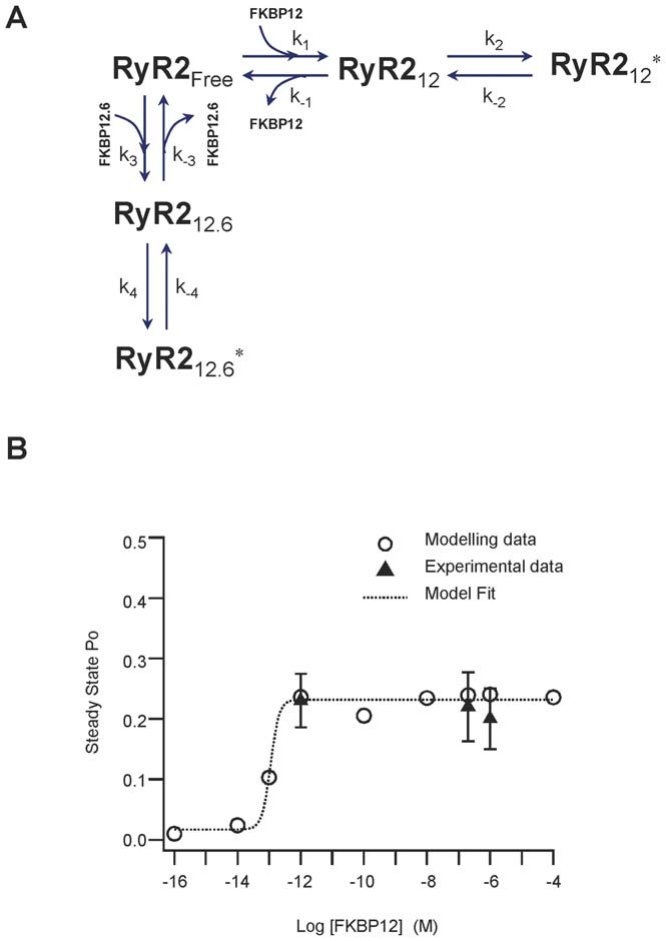
A mechanism to describe the influence of FK-binding proteins on RyR2 gating. (A) Proposed schematic representation of the interactions of FK-binding proteins with RyR2. RyR2_Free_ represents the RyR2 channel state when no FK-binding proteins are bound. RyR2_12_ and RyR2_12.6_ represent the states of the channel following the binding of either FKBP12 or FKBP12.6. RyR2_12_^*^ and RyR2_12.6_^*^ represent the altered conformations of RyR2 that result from the tight binding to FKBP12 or FKBP12.6, respectively, and give rise to a change in the Ca^2+^-sensitivity of RyR2. (B) Model derived FKBP12 concentration-response relationship showing RyR2 Po following addition of various concentrations of FKBP12. Steady state Po values were computed by taking a trial average over 100 Monte Carlo simulations of a single channel (circles). According to the fit, the model predicts the EC_50_ for FKBP12 = 0.12 pM. As a comparison, the mean Po values obtained from the experimental data at 1 pM, 200 nM and 1 µM FKBP12 have also been plotted (triangles; error bars are SEM) Parameter values used in the simulations are given in Table 1.

We constructed a kinetic model to describe the possible channel states and the transitions between states that arise from the binding of FKBP12 or FKBP12.6 to RyR2. We then used this model to simulate our data. We assume the simplest case; that RyR2 can exist in the FKBP-free state (RyR2_Free_) and that it can bind FKBP12 or FKBP12.6 forming either the FKBP12 bound state (RyR2_12_) or the FKBP12.6 bound state (RyR2_12.6_). Each binding event leads to a conformational change in RyR2, which in turn modifies channel function in a manner specific to the isoform of FKBP that is bound. Thus, when FKBP12 is bound, the Ca^2+^-sensitivity of RyR2 is increased (state RyR2_12*_), whereas when FKBP12.6 is bound, any change in Ca^2+^-sensitivity is much less marked (state RyR2_12.6*_). The model is shown in Figure 6A.

From this model, we derived equations using mass-action kinetics for the transitions between the various states as shown below:
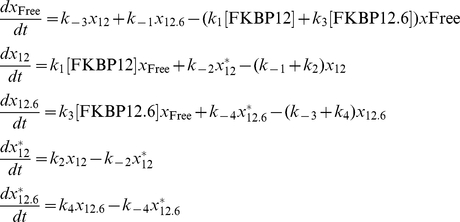
where the probability of the channel dwelling in the state RyR2_i_ is denoted by *x*_i_, where i = Free, 12, 12.6, 12^*^, 12.6^*^.

Equilibrium approximation with respect to FKBP12 and FKBP12.6 binding and the subsequent change in Ca^2+^-sensitivity of RyR2 yields:
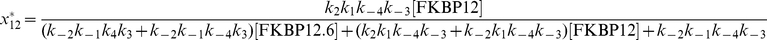


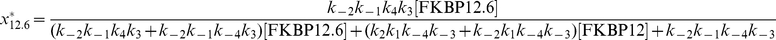
We modelled the stochastic gating of RyR2 as a continuous-time Markov process by considering the simplest transition-state diagram that can describe the kinetics of RyR2 gating between the closed (C) and open (O) states,
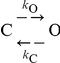
where k_O_ is the rate (probability) of transition from state C to state O and k_C_ is the rate (probability) of the reverse transition.

In our model, the rate k_O_ depends on the probability that RyR2 is bound to FKBP12 or to FKBP12.6. Of course, the Po of RyR2 channels also depends on other factors such as the cytosolic and luminal [Ca^2+^], on the binding of other closely associated proteins and on the redox and phosphorylation states of the channels. We assume, however, that these factors are maintained constant throughout each experiment since Po remains stable (although gating is modal [32], [35]) over time until the addition of FKBP12 or FKBP12.6. We therefore include a constant (*α*), that accounts for this spontaneous background RyR2 activity. Thus, in the presence of FK-binding proteins, RyR2 Po is given by the sum of the probabilities that RyR2 exists in the two possible open states, namely *x*_12_^*^+*x*_12.6_^*^, i.e.
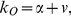
(1)where, *ν* = *x*_12_^*^+*x*_12.6_^*^. The parameter values of the model are given in Table 1.

**Table 1:** Parameter values of the RyR2 model.

Parameter values of the RyR2 model	
*k*_C_ = 480 s^−1^	*α* = 0.005
*k*_1_ = 0.29×10^2^ µM^−1^ s^−1^	*k*_−1_ = 6.11×10^−6^ s^−1^
*k*_2_ = 0.03 s^−1^	*k*_−2_ = 0.18 s^−1^
*k*_3_ = 0.33×10^6^ µM^−1^ s^−1^	*k*_−3_ = 4.2×10^−4^ s^−1^
*k*_4_ = 0.001 s^−1^	*k*_−4_ = 0.25 s^−1^

**Figure 7:**
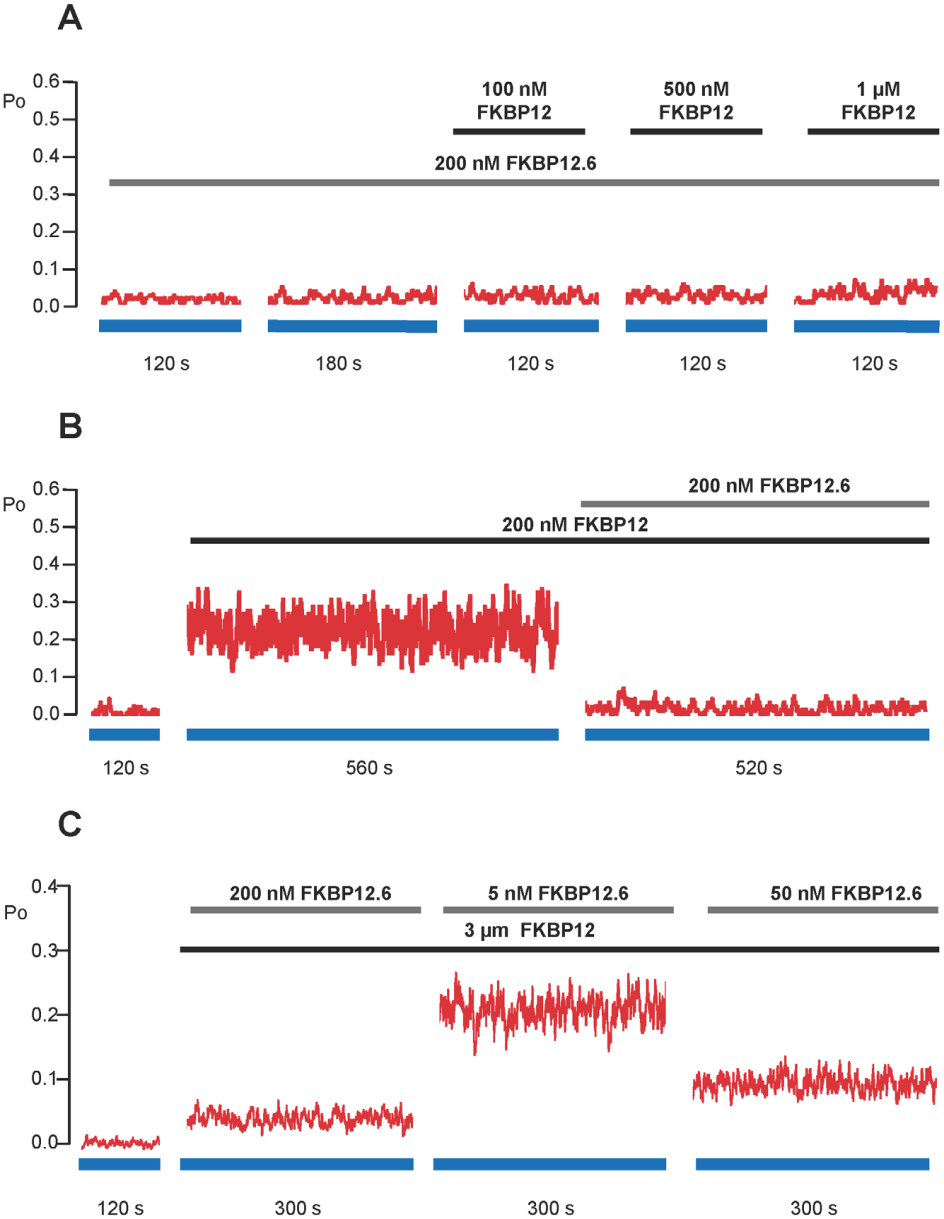
Model predictions of RyR2 Po at equilibrium in the presence of both FKBP12 and FKBP12.6. Model predictions of RyR2 equilibrium Po for the combinations of FKBP12 and FKBP12.6 that are present in the experiments detailed in Figure 4. Panels A and B show the predicted Po at equilibrium of a typical RyR2 channel under the experimental conditions shown in Figures 4A and 4B, respectively. Whereas the single-channel experiments (Figure 4) which, on average, run for approximately 20 min, typically reflect the effects of the protein that bound first (since dissociation rates of FKBP12 and 12.6 are so slow), the model allows us to predict what would happen at equilibrium. (C) Model predictions of equilibrium RyR2 Po if FKBP12.6 levels vary while FKBP12 is maintained at a physiological level of 3 µM. Steady-state Po values at equilibrium have been computed by taking a trial average over 100 Monte Carlo simulations of a single RyR2 channel. The parameter values used in the simulations are given in Table 1.

Parameter values used in the model simulations shown in Figures 6 and 7. We used a previously published estimate of the rate of dissociation of FKBP12.6 from RyR2 (*k*_−3_ = 4.2×10^−4^ s^−1^) [15] but all other values were were chosen to ensure that the model reproduces all our experimental data. The value of *k*_O_ is given by Equation (1).

The model described above allows us to compute the average RyR2 Po for various FK-binding protein concentrations and under different conditions such as the sequential addition of FKPB12 following FKBP12.6 and *vice versa*. In choosing parameter values for the model, as a starting point we used published estimates of the rate constants of association and dissociation of FKBP12 and FKBP12.6 to RyR2 [15]. However, in order to reproduce the FKBP12 concentration-dependence of RyR2 Po that we observed experimentally together with the obvious slow dissociation of FKBP12 from RyR2, we found that we needed to change the binding rates of FKBP12. In this way, we calibrated the model parameters that are related to the effects of FKBP12 on RyR2 Po. Figure 6B illustrates the close match between the simulated data (circles) and the experimental data (triangles).

Our model also allows us to predict how RyR2 gating in the steady-state would be affected if the experimental protocols shown in Figures 4A and 4B were applied but the binding of FKBP12 and FKBP12.6 to RyR2 was allowed to reach equilibrium. This is useful because the slow dissociation rates of FKBP12 and FKBP12.6 from RyR2 impose the requirement for hours of steady-state single-channel recording (for each single-channel) before a true steady-state equilibrium Po could be calculated. Figures 7A and 7B predict the equilibrium gating of RyR2 expected under the experimental conditions of Figure 4A and 4B, respectively.

It has been suggested that there is a reduced amount of FKBP12.6 bound to RyR2 channels in heart failure [5], [42], [43] and that this leads to an increased tendency for SR Ca^2+^-release events during diastole and an increased likelihood of developing arrhythmias. There are many mechanisms by which this could occur but one possibility would be that there is simply less FKBP12.6 expressed in the cardiac cells in heart failure. Our model can predict how changes in cellular FKBP12.6 levels could affect RyR2 Po at equilibrium if FKBP12 remained at physiological levels (3 µM). We do not know the physiological level of FKBP12.6 in a cardiac cell except that it is below detection levels and therefore much lower than the micromolar levels of FKBP12. Figure 7C predicts the RyR2 Po at equilibrium for three different concentrations of FKBP12.6 given that FKBP12 levels remain at 3 µM. The model indicates that RyR2 Po at equilibrium would rise in a concentration-dependent manner as the levels of FKBP12.6 were reduced.

### Effects of FK-binding proteins on waves of CICR in cardiac cells

**Figure 8:**
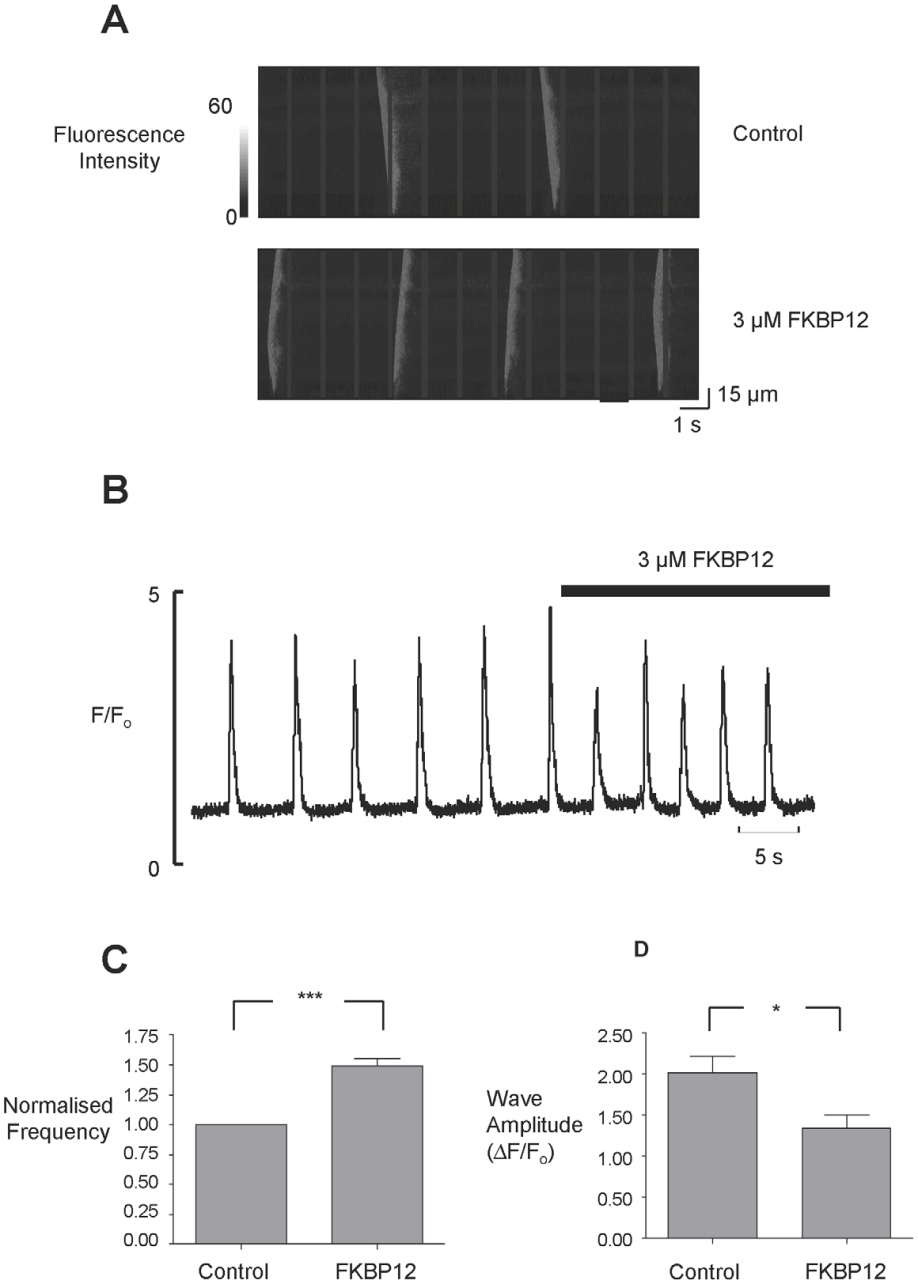
FKBP12 causes an increase in Ca^2+^-wave frequency. (A) Linescan images collected from permeabilised cardiac myocytes under control conditions (top), and in the presence of 3 µM FKBP12 (below). (B) Mean fluorescence records from the linescans shown in panel A. (C) illustrates the mean change in frequency caused by 3 µM FKBP12 (SEM; n = 14; ***p≤0.001) and (D) shows the effect of 3 µM FKBP12 on wave amplitude (SEM; n = 14; *p = 0.028).

Although the unresolved controversy over the single-channel effects of FKBP12.6 [3], [8], [10], [11] is very noticeable in the literature, there is general agreement with the idea that FKBP12.6 can ‘stabilize’ or reduce cardiac SR Ca^2+^-release under pathological conditions. In heart failure, for example, there is an increased tendency for the generation of spontaneous waves of SR Ca^2+^-release and the development of delayed after depolarisations (DADs) [44]–[48]. Our single-channel experiments, where the endogenous FKBP's were displaced prior to experimentation, demonstrate the effects of FKBP12 and FKBP12.6 on the gating of RyR2. These data now suggest a possible mechanism by which FKBP12.6 could act as a ‘stabilizer’ of SR Ca^2+^-release in cardiac cells. FKBP12 would be expected to sensitise RyR2 to a rise in cytosolic Ca^2+^ thereby potentiating CICR. FKBP12.6 would antagonise this action. We therefore investigated if FKBP12 could augment spontaneous waves of CICR in rat permeabilised cardiac myocytes and whether FKBP12.6 could antagonise this effect. At 5 minutes post-permeabilisation, cells were superfused with Fluo-5F and 234 nM Ca^2+^. Figure 8A (top trace) shows the typical Ca^2+^-waves under these conditions. We then perfused with a physiological concentration of FKBP12 (3 µM) (bottom trace) and this caused an increase in wave frequency. Figure 8B shows the mean fluorescence records (measured from the linescans in panel A) demonstrating that FKBP12 not only increases the frequency of Ca^2+^-waves but also decreases wave amplitude. Figure 8C and D illustrate the mean wave frequency and mean wave amplitude before and after superfusion with FKBP12. These results are exactly what we would expect if RyR2 Po is increased, as similar effects are observed with other agents, such as caffeine, that increase RyR2 Po [49].

**Figure 9:**
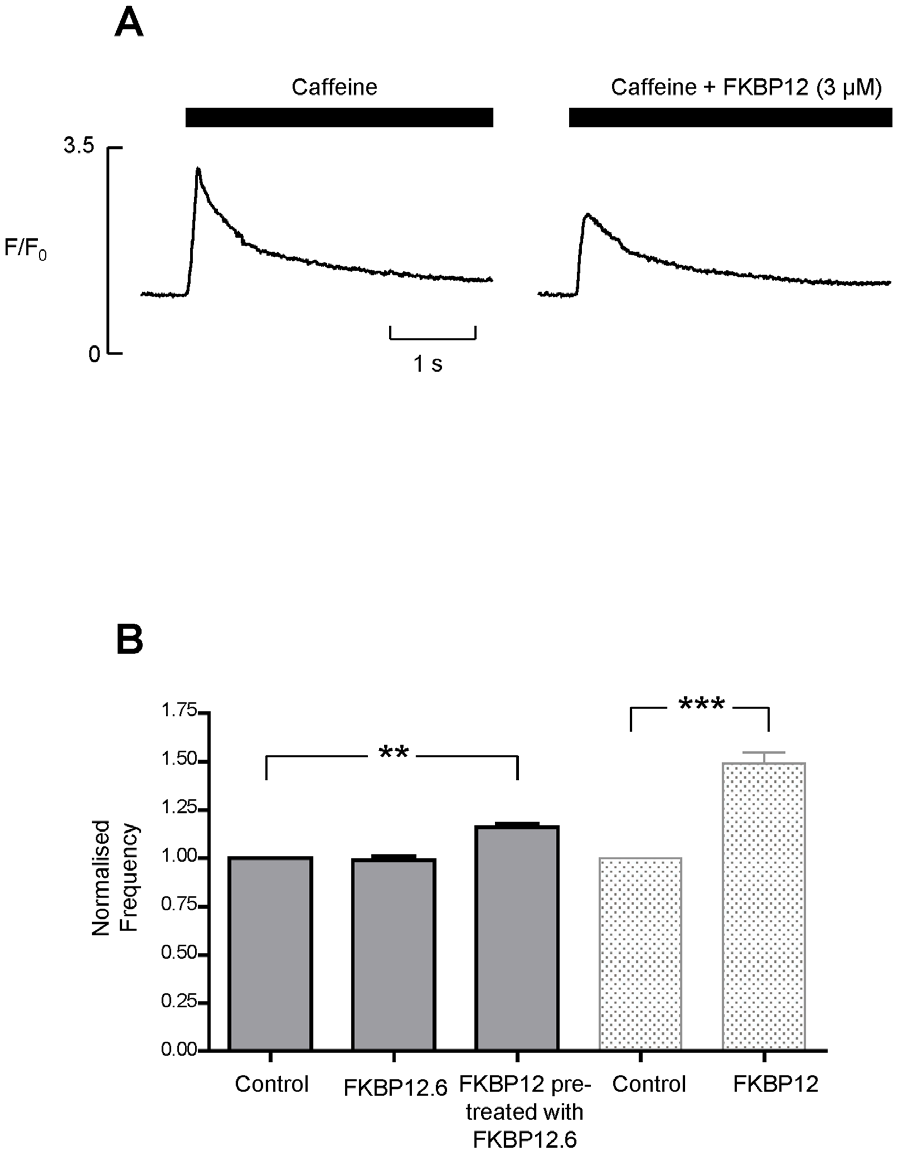
FKBP12 (3 µM) reduces SR Ca^2+^-content. (A) Typical changes in fluorescence following caffeine addition are shown for the same cell in control conditions (left) and in the presence of FKBP12 (3 µM) (right). Caffeine (20 mM) was applied for the period shown by the solid bars. (B) shows the effects of FKBP12.6 (200 nM) on wave frequency. Data from Figure 8C (rightmost two bars) is included so that the effects of FKBP12 in the absence and presence of added FKBP12.6 can be directly compared. Pre-treatment with 200 nM FKBP12.6 reduces the effect of FKBP12 (3 µM) on Ca^2+^- wave frequency. FKBP12.6 (200 nM) alone has no effect on wave frequency. The FKBP12.6 percentage increase in wave frequency = 16.25±0.021% (SEM; n = 5; **p = 0.002). The FKBP12 percentage increase in wave frequency = 50±0.058% (SEM; n = 14; ***p≤0.001). Values are normalised to the relevant control.

The reduced wave amplitude may result because there is a decrease in the SR Ca^2+^-content. We examined this by applying 20 mM caffeine to release SR Ca^2+^ before and after perfusion with 3 µM FKBP12. Figure 9A demonstrates that 20 mM caffeine produced a larger Ca^2+^-transient in control (3.2±0.1 ΔF/Fo) than in the presence of FKBP12 (2.5±0.2; n = 6; *p*<0.003) indicating that there is a reduced SR Ca^2+^-content in the presence of FKBP12.

Our single-channel experiments suggest that FKBP12.6 behaves as an antagonist of the actions of FKBP12 on RyR2. To investigate if this mechanism is relevant to SR Ca^2+^-release in cardiac cells we perfused cells with FKBP12.6. The levels of FKBP12.6 in cardiac cells are so low that they are undetectable [2], [19], hence, unlike FKBP12, we do not know the physiological concentration. We therefore used 200 nM FKBP12.6, a concentration higher than that likely to be present in a cell. This concentration of FKBP12.6 did not produce any significant changes in wave frequency as shown in Figure 9B. To test the ability of FKBP12.6 to antagonise FKBP12, we first pre-incubated the cells with 200 nM FKBP12.6 and then perfused with FKBP12 (3 µM) in the presence of 200 nM FKBP12.6. Comparison of the data in Figure 8C with that in Figure 9B shows that although FKBP12 still significantly increases wave frequency, pre-incubation with FKBP12.6 reduces the extent of this effect (FKBP12 alone increases wave frequency by 50±0.06% (SEM; n = 14; *p*≤0.001) but after pre-incubation with FKBP12.6, FKBP12 only increases wave frequency by 16.25±0.02% (SEM; n = 5; *p* = 0.002)).

## Discussion

We demonstrate that FKBP12 can activate single RyR2 channels at very low concentrations. Like many other activators of RyR channels including Ca^2+^, FKBP12 is a partial agonist. We also show that FKBP12.6 has no intrinsic ability to reduce RyR2 Po but perhaps may have extremely low efficacy as an activator of RyR2. It is, however, able to antagonise the effects of FKBP12. These results suggest that FKBP12 and FKBP12.6 may compete for the same binding sites on RyR2, especially since both molecules are structurally so similar. We cannot rule out the possibility that FKBP12.6 acts to reduce FKBP12-induced increases in Po by binding at distinct sites although this seems unlikely since FKBP12.6 does not, alone, reduce the Ca^2+^-activated openings. There has been rather an over-emphasis of the exclusive nature of FKBP12.6 binding to RyR2 and the possibility that FKBP12 may affect RyR2 function has been somewhat overlooked. Our results should not be too unexpected however, since Jeyakumar et al. (2001) have previously suggested that FKBP12 binds to the cardiac isoform of many species although they also indicate that it may not bind to canine RyR2. Seidler et al. (2007) have also demonstrated binding of GST-FKBP12 to RyR2. Our reductionist approach has revealed new mechanisms underlying the functional interactions of FKBP12 and FKBP12.6 with RyR2. This new information may help to resolve the controversies surrounding the effects of FKBP12.6 on RyR2 channel gating and the many descriptions of the cardiac ‘stabilizing’ properties of FKBP12.6. For example, in models of heart failure, it has been reported that over-expressing FKBP12.6 [14], [16] or introducing molecules of FKBP12.6 that may bind tighter to RyR2 [50], [51], have beneficial effects. Our results suggest that the beneficial effects of these interventions is not due to the direct inhibitory action of FKBP12.6, but to the ability of FKBP12.6 to antagonise the action of FKBP12 as an activator of RyR2.

The slow rate of dissociation of FKBP12 from RyR2, evidenced by the washout experiments (Figure 3), means that the binding of FKBP12 to RyR2 is practically irreversible on the timescale of a single-channel experiment. This explains the shape of the concentration-response relationship (Figure 2C) and the high-affinity of FKBP12 for RyR2. It is thought that there are 4 FKBP binding sites on each RyR tetramer [2], [52] yet it is not known how many binding sites must be occupied for a functional response. Our own results also do not provide any clues about the number of FKBP12 molecules required for an increase in Po. One aspect to be aware of is that it is very difficult to be sure that *all* the endogenous FKBP has been removed before adding back FKBP to observe its effect. In general, depletion of FKBP means simply ‘below the detection level of a Western blot’ rather than complete removal of the protein. This is also true for our cardiac membrane preparations. Although we can ‘deplete’ our cardiac SR of FKBP with a high salt solution we cannot definitively prove that we have removed every molecule. However, since exogenously added FKBP12 always causes a consistent increase in Po, it is clear that, in our experiments, there are vacant binding sites on RyR2 for FKBPs that appear to be functionally important. Many studies have used compounds such as FK506 or rapamycin to lower the levels of FKBPs present in cells or isolated membrane vesicles. There have been reports, however, that FK506 [53] and rapamycin [29] directly affect RyR1 gating. We also find that rapamycin causes very marked activation of RyR2 channels that appears to be unrelated to the removal of FKBPs (results not shown) and therefore we have not used these drugs in our study. Thus, the frequent use of such drugs may have confused our understanding of the functional effects of FKBPs on RyR channel gating, adding fuel to any controversy.

We found that the main effect of FKBP12 was to increase the frequency of channel openings. FKBP12 can also increase the duration of channel openings but this effect was less pronounced, probably playing a greater role in the presence of other channel activators. Since the mechanism by which cytosolic Ca^2+^ activates RyR2 is primarily by increasing the frequency of channel opening with only a small lengthening of open lifetime durations [35], [54] it is likely that FKBP12 is sensitising RyR2 to cytosolic Ca^2+^. Indeed, the Po of channels that have been activated by FKBP12 can be reduced to zero when cytosolic Ca^2+^ is reduced to sub-activating levels providing strong evidence for this mechanism of action.

This mechanism of action of FKBP12 is important to consider when looking at how FKBP12 would influence the opening of RyR2 channels *in situ*. In a cardiac cell, we might predict that FKBP12 would increase the frequency of channel openings during diastole; a time when RyR2 Po is normally low. Essentially, FKBP12 would sensitise RyR2 channels to the effects of small rises in cytosolic Ca^2+^ and the channels would become ‘leaky’ during diastole. This mechanism also provides the best explanation for the FKBP12-induced increased frequency of spontaneous Ca^2+^-waves that we observe in our permeabilised cell experiments. The increased wave frequency is associated with a lower SR Ca^2+^-content as shown by Figure 9A. Other activators of RyR2, for example, caffeine, are also known to increase the frequency of spontaneous release events and decrease SR Ca^2+^-content [49]. This is expected since the SR will be depleted of Ca^2+^ following a spontaneous wave of CICR and the SR must refill before another wave is possible [27], [55]. Normally a wave would not be initiated before the SR Ca^2+^-content was sufficiently recovered. If, however, RyR2 was sensitised to the effects of cytosolic Ca^2+^ (for example, by caffeine or FKBP12), then a wave of CICR would be triggered at a lower SR Ca^2+^-content, reducing the requirement for the SR to fill. The lower SR Ca^2+^-content would give rise to smaller waves and since less Ca^2+^ is released with each wave, the SR would need less time to refill and so the waves would be more frequent. Thus, our results can be explained by an effect on RyR2 gating.

Two previous studies have investigated how FKBP12 may affect SR Ca^2+^-release in permeabilised cardiac cells [15], [56] but one study found a decrease in Ca^2+^-spark amplitude and width after perfusion with 1 µM [15] whereas the other study found an increase in Ca^2+^-spark amplitude and width after over-expression of FKBP12 [56]. These seemingly opposite results highlight the complex nature of the interactions of FKBPs with RyR2 and perhaps are due to the particular conditions of the experiments. For example, Guo et al. (2010) used a fluorescent FKBP12 molecule and the large structural addition may have affected the functional interaction with RyR2. Seidler et al. (2007) over-expressed FKBP12 and therefore before cell permeabilisation it would be expected that a greater than physiological level of FKBP12 would bind to RyR2 channels. After cell permeabilisation, since there was no added FKBP in the perfusate, it would be expected that there would be a lower than physiological level of FKBP12 molecules associated with RyR2 since FKBP12 would be lost in the perfusate. In the light of our results, it is interesting to consider how endogenous FKBP12 would influence RyR2 behaviour in the FKBP12.6 knockout mouse model [17]. Ca^2+^-spark amplitude and duration are increased in cardiac cells isolated from the FKBP12.6 knockout and it is suggested that the RyR channels are open for longer [17]. This is what might be expected if the activatory effects of FKBP12 were left unconstrained by the antagonistic effects of FKBP12.6.

**Figure 10:**
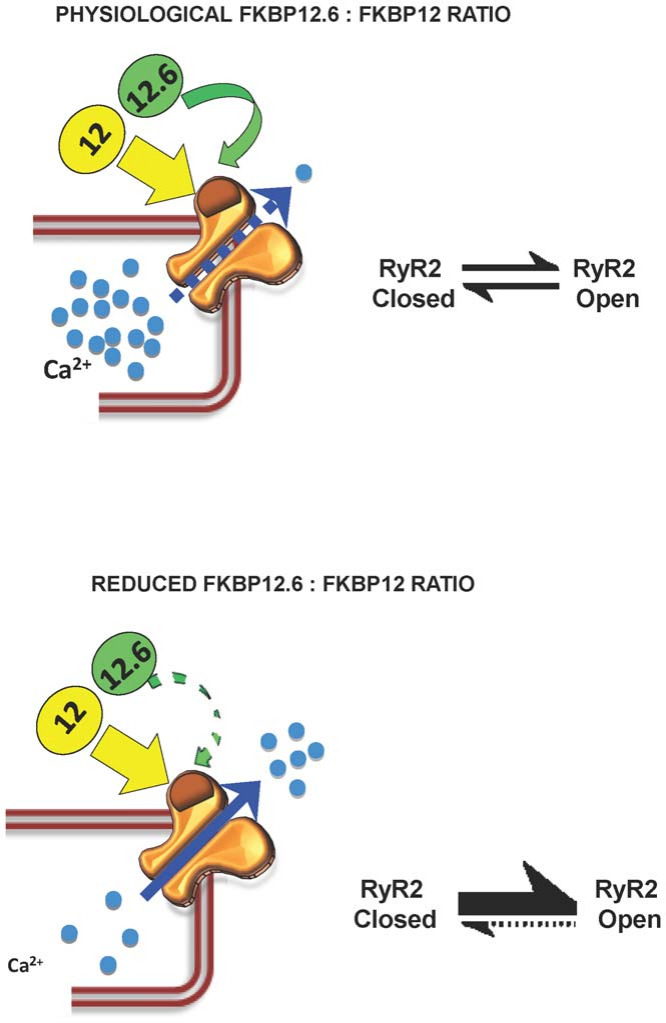
Model of proposed dual regulation of cardiac SR Ca^2+^-release by FKBP12 and FKBP12.6. (A) Under normal physiological conditions, the binding of FKBP12.6 to RyR2 provides sufficient restraint on the stimulatory effects of FKBP12 to allow excitation-contraction coupling to proceed without undue risk of arrhythmia generation caused by diastolic leak of intracellular Ca^2+^. (B) If the binding of FKBP12.6 to RyR2 is impaired in disease, for example, because of under expression of FKBP12.6 leading to a reduced ratio of FKBP12.6: FKBP12 in the cell (or due a conformational change to RyR2) then the effects of FKBP12 may dominate. This could cause increased sensitivity of RyR2 to cytosolic Ca^2+^, an increased Po and increased diastolic leak of intracellular Ca^2+^.

Our results suggest that the relative levels of FKBP12 and FKBP12.6 in a cardiac cell will be important in the regulation of SR Ca^2+^-release since FKBP12.6 can inhibit the effects of FKBP12. This is confirmed by mathematical modelling of our data (Figure 7C). For example, any lowering in the expression levels of FKBP12.6 may swing the balance of FKBP12/FKBP12.6 binding to RyR2 to a level where RyR2 sensitivity for cytosolic Ca^2+^ is increased. This would promote a greater tendency for the development of spontaneous waves of CICR and delayed after depolarisations (DADs) and an increased likelihood of fatal arrhythmias. A model of this scheme is shown in Figure 10. There has been much controversy over the idea that FKBP12.6 acts as a natural ‘stabiliser’ of RyR2 activity in cardiac cells and that in heart failure there is a lower level of FKBP12.6 associated with RyR2 [5], [11], [43], [57]–[59]. Much of the controversy arose because many groups, including our own, were not able to find any evidence that FKBP12.6 could reduce the Po of RyR2. Our work now reconciles these apparent inconsistencies and shows that FKBP12.6 can lower RyR2 Po indirectly by antagonising the activating effects of FKBP12. The many cellular changes that occur in heart failure could lead to changes in the ratio of FKBP12.6/FKBP12 levels in cardiac cells or to conformational changes to RyR2 that affect the relative affinity/efficacy of FKBP12 or FKBP12.6. Such changes could directly impinge on SR Ca^2+^-release and could lead to the ‘leaky’ RyR2 channels, reduced SR Ca^2+^-load and increased likelihood of arrhythmias that occur in heart failure.

## Supporting Information

Figure S1 **The effects of high salt incubation on the association of FKBPs with cardiac mixed membrane (MM) vesicles.** Western blot analysis following incubation of cardiac mixed membrane vesicles with a high sucrose (0.4 M) solution (left lane) or a high salt (0.4 M) solution (right lane). Vesicles were loaded at 100 µg. Western blots were probed with an anti-FKBP12 antibody (recognising both FKBP12 and FKBP12.6). Size markers are indicated in kDa.
(TIFF)

## References

[1] Valdeolmillos M, O'Neill SC, Smith GL, Eisner DA (1989). Calcium-induced calcium-release activates contraction in intact cardiac cells.. Pflug Arch Eur J Phy.

[2] Timerman AP, Onoue H, Xin HB, Barg S, Copello J (1996). Selective binding of FKBP12.6 by the cardiac ryanodine receptor.. J Biol Chem.

[3] Lam E, Martin MM, Timerman AP, Sabers C, Fleischer S (1995). A novel FK506 binding protein can mediate the immunosuppressive effects of FK506 and is associated with the cardiac ryanodine receptor.. J Biol Chem.

[4] Xin HB, Rogers K, Qi Y, Kanematsu T, Fleischer S (1999). Three amino acid residues determine selective binding of FK506-binding protein 12.6 to the cardiac ryanodine receptor.. J Biol Chem.

[5] Marx SO, Reiken S, Hisamatsu Y, Jayaraman T, Burkhoff D (2000). PKA phosphorylation dissociates FKBP12.6 from the calcium release channel (ryanodine receptor): Defective regulation in failing hearts.. Cell.

[6] Wehrens XH, Lehnart SE, Huang F, Vest JA, Reiken SR (2003). FKBP12.6 deficiency and defective calcium release channel (ryanodine receptor) function linked to exercise-induced sudden cardiac death.. Cell.

[7] Wehrens XH, Lehnart SE, Reiken S, van der NR, Morales R (2005). Enhancing calstabin binding to ryanodine receptors improves cardiac and skeletal muscle function in heart failure.. Proc Natl Acad Sci U S A.

[8] Marx SO, Gaburjakova J, Gaburjakova M, Henrikson C, Ondrias K (2001). Coupled gating between cardiac calcium release channels (ryanodine receptors).. Circ Res.

[9] Kaftan E, Marks AR, Ehrlich BE (1996). Effects of rapamycin on ryanodine receptor/Ca^2+^-release channels from cardiac muscle.. Circ Res.

[10] Barg S, Copello JA, Fleischer S (1997). Different interactions of cardiac and skeletal muscle ryanodine receptors with FK-506 binding protein isoforms.. Am J Physiol.

[11] Xiao JM, Tian XX, Jones PP, Bolstad J, Kong HH (2007). Removal of FKBP12.6 does not alter the conductance and activation of the cardiac ryanodine receptor or the susceptibility to stress-induced ventricular arrhythmias.. J Biol Chem.

[12] Stewart R, Song L, Carter SM, Sigalas C, Zaccai NR (2008). Single-channel characterization of the rabbit recombinant RyR2 reveals a novel inactivation property of physiological concentrations of ATP.. J Membr Biol.

[13] Xiao RP, Valdivia HH, Bogdanov K, Valdivia C, Lakatta EG (1997). The immunophilin FK506-binding protein modulates calcium release channel closure in rat heart.. J Physiol.

[14] Loughrey CM, Seidler T, Miller SL, Prestle J, MacEachern KE (2004). Over-expression of FK506-binding protein FKBP12.6 alters excitation-contraction coupling in adult rabbit cardiomyocytes.. J Physiol.

[15] Guo T, Cornea RL, Huke S, Camors E, Yang Y (2010). Kinetics of FKBP12.6 binding to ryanodine receptors in permeabilized cardiac myocytes and effects on Ca^2+^ sparks.. Circ Res.

[16] Prestle J, Janssen PM, Janssen AP, Zeitz O, Lehnart SE (2001). Overexpression of FK506-binding protein FKBP12.6 in cardiomyocytes reduces ryanodine receptor-mediated Ca^2+^ leak from the sarcoplasmic reticulum and increases contractility.. Circ Res.

[17] Xin HB, Senbonmatsu T, Cheng DS, Wang YX, Copello JA (2002). Oestrogen protects FKBP12.6 null mice from cardiac hypertrophy.. Nature.

[18] Shou W, Aghdasi B, Armstrong DL, Guo Q, Bao S (1998). Cardiac defects and altered ryanodine receptor function in mice lacking FKBP12.. Nature.

[19] Timerman AP, Wiederrecht G, Marcy A, Fleischer S (1995). Characterization of an exchange reaction between soluble FKBP-12 and the FKBP-ryanodine receptor complex. Modulation by FKBP mutants deficient in peptidyl-prolyl isomerase activity.. J Biol Chem.

[20] Chelu MG, Danila CI, Gilman CP, Hamilton SL (2004). Regulation of ryanodine receptors by FK506 binding proteins.. Trends Cardiovasc Med.

[21] Deivanayagam CCS, Carson M, Thotakura A, Narayana SVL, Chodavarapu RS (2000). Structure of FKBP12.6 in complex with rapamycin.. Acta Crystallogr.

[22] Fulton KF, Jackson SE, Buckle AM (2003). Energetic and structural analysis of the role of tryptophan 59 in FKBP12.. Biochemistry (Mosc).

[23] Sitsapesan R, Montgomery RAP, MacLeod KT, Williams AJ (1991). Sheep cardiac sarcoplasmic reticulum calcium release channels: modification of conductance and gating by temperature.. J Physiol.

[24] Colquhoun D, Sigworth FJ, Sakmann B, Neher E (1983). Fitting and statistical analysis of single-channel recording.. Single-channel recording.

[25] Sigworth FJ, Sine SM (1987). Data transformations for improved display and fitting of single-channel dwell time histograms.. Biophys J.

[26] Eisner DA, Nichols CG, O'Neill SC, Smith GL, Valdeolmillos M (1989). The effects of metabolic inhibition on intracellular calcium and pH in isolated rat ventricular cells.. J Physiol.

[27] Smith GL, O'Neill SC (2001). A comparison of the effects of ATP and tetracaine on spontaneous Ca^2+^ release from rat permeabilised cardiac myocytes.. J Physiol.

[28] Noble JE, Bailey MJA, Richard RB, Murray PD (2009). Chapter 8 Quantitation of Protein.. Methods Enzymol: Academic Press.

[29] Ahern GP, Junankar PR, Dulhunty AF (1997). Ryanodine receptors from rabbit skeletal muscle are reversibly activated by rapamycin.. Neurosci Lett.

[30] Ahern GP, Junankar PR, Dulhunty AF (1997). Subconductance States in Single-channel Activity of Skeletal Muscle Ryanodine Receptors After Removal of FKBP12.. Biophys J.

[31] Laver DR, Lamb GD (1998). Inactivation of Ca^2+^ release channels (ryanodine receptors RyR1 and RyR2) with rapid steps in Ca^2+^ and voltage.. Biophys J.

[32] Zahradnikova A, Dura M, Gyorke S (1999). Modal gating transitions in cardiac ryanodine receptors during increases of Ca^2+^ concentration produced by photolysis of caged Ca^2+^.. Pflug Arch Eur J Phy.

[33] Sitsapesan R, Williams AJ (1994). Regulation of the gating of the sheep cardiac sarcoplasmic reticulum Ca^2+^ -release channel by luminal Ca^2+^.. J Membr Biol.

[34] Copello JA, Barg S, Onoue H, Fleischer S (1997). Heterogeneity of Ca^2+^ gating of skeletal muscle and cardiac ryanodine receptors.. Biophys J.

[35] Saftenku E, Williams AJ, Sitsapesan R (2001). Markovian Models of Low and High Activity Levels of Cardiac Ryanodine Receptors.. Biophys J.

[36] Wagenknecht T, Radermacher M, Grassucci R, Berkowitz J, Xin HB (1997). Locations of calmodulin and FK506-binding protein on the three-dimensional architecture of the skeletal muscle ryanodine receptor.. J Biol Chem.

[37] Sharma MR, Jeyakumar LH, Fleischer S, Wagenknecht T (2006). Three-dimensional visualization of FKBP12.6 binding to an open conformation of cardiac ryanodine receptor.. Biophys J.

[38] Samso M, Shen XH, Allen PD (2006). Structural characterization of the RyR1-FKBP12 interaction.. J Mol Biol.

[39] Carter S, Pitt SJ, Colyer J, Sitsapesan R (2011). Ca^2+^-dependent phosphorylation of RyR2 can uncouple channel gating from direct cytosolic Ca^2+^ regulation.. J Membr Biol.

[40] Sigalas C, Bent S, Kitmitto A, O'Neill S, Sitsapesan R (2009). Ca^2+^-calmodulin can activate and inactivate cardiac ryanodine receptors.. Br J Pharmacol.

[41] Sitsapesan R, Williams AJ (1990). Mechanisms of caffeine activation of single calcium-release channels of sheep cardiac sarcoplasmic reticulum.. J Physiol.

[42] Lehnart SE, Wehrens XH, Marks AR (2004). Calstabin deficiency, ryanodine receptors, and sudden cardiac death.. Biochem Biophys Res Commun.

[43] Huang F, Shan J, Reiken S, Wehrens XH, Marks AR (2006). Analysis of calstabin2 (FKBP12.6)-ryanodine receptor interactions: rescue of heart failure by calstabin2 in mice.. Proc Natl Acad Sci U S A.

[44] Pogwizd SM, Bers DM (2004). Cellular Basis of Triggered Arrhythmias in Heart Failure.. Trends Cardiovasc Med.

[45] Eisner DA, Kashimura T, O'Neill SC, Venetucci LA, Trafford AW (2009). What role does modulation of the ryanodine receptor play in cardiac inotropy and arrhythmogenesis?. J Mol Cell Cardiol.

[46] Venetucci LA, Trafford AW, O'Neill SC, Eisner DA (2008). The sarcoplasmic reticulum and arrhythmogenic calcium release.. Cardiovasc Res.

[47] Ng GA, Cobbe SM, Smith GL (1998). Non-uniform prolongation of intracellular Ca^2+^ transients recorded from the epicardial surface of isolated hearts from rabbits with heart failure.. Cardiovasc Res.

[48] Sobie EA, Guatimosim S, Gómez-Viquez L, Song L-S, Hartmann H (2005). The Ca^2+^ leak paradox and “rogue ryanodine receptors”: SR Ca^2+^ efflux theory and practice.. Prog Biophys Mol Biol.

[49] Trafford AW, Sibbring GC, Diaz ME, Eisner DA (2000). The effects of low concentrations of caffeine on spontanteous Ca^2+^ release in isolated rat ventricular myocytes.. Cell Calcium.

[50] Seidler T, Teucher N, Hellenkamp K, Unsöld B, Grebe C (2011). Limitations of FKBP12.6-directed treatment strategies for maladaptive cardiac remodeling and heart failure.. J Mol Cell Cardiol.

[51] Huang F, Shan J, Reiken S, Wehrens XH, Marks AR (2006). Analysis of calstabin2 (FKBP12.6)-ryanodine receptor interactions: rescue of heart failure by calstabin2 in mice.. Proc Natl Acad Sci U S A.

[52] Jeyakumar LH, Ballester L, Cheng DS, McIntyre JO, Chang P (2001). FKBP binding characteristics of cardiac microsomes from diverse vertebrates.. Biochem Biophys Res Commun.

[53] Ahern GP, Junankar PR, Dulhunty AF (1994). Single channel activity of the ryanodine receptor calcium release channel is modulated by FK-506.. FEBS Lett.

[54] Sitsapesan R, Williams AJ (1994). Gating of the native and purified cardiac SR Ca^ 2+^ -release channel with monovalent cations as permeant species.. Biophys J.

[55] Overend CL, Eisner DA, O'Neill SC (1997). The effect of tetracaine on spontaneous Ca^2+^ release and sarcoplasmic reticulum calcium content in rat ventricular myocytes.. J Physiol.

[56] Seidler T, Loughrey CM, Zibrova D, Kettlewell S, Teucher N (2007). Overexpression of FK-506 binding protein 12.0 modulates excitation contraction coupling in adult rabbit ventricular cardiomyocytes.. Circ Res.

[57] Marks AR (2000). Cardiac intracellular calcium release channels - Role in heart failure.. Circ Res.

[58] Valdivia HH (2001). Cardiac Ryanodine Receptors and Accessory Proteins. Augmented Expression Does Not Necessarily Mean Big Function.. Circ Res.

[59] Valdivia HH, Jiang MT, Lokuta AJ, Farrell EF, Wolff MR (2002). Abnormal Ca^2+^ release, but normal ryanodine receptors, in canine and human heart failure.. Circ Res.

