# Supplementary figures and images for: FKBP12 Activates the Cardiac Ryanodine Receptor Ca2+-Release Channel and Is Antagonised by FKBP12.6
Source: PLoS One. 2012 Feb 21;7(2):e31956. doi: 10.1371/journal.pone.0031956 (PMC3283708; doi:10.1371/journal.pone.0031956)

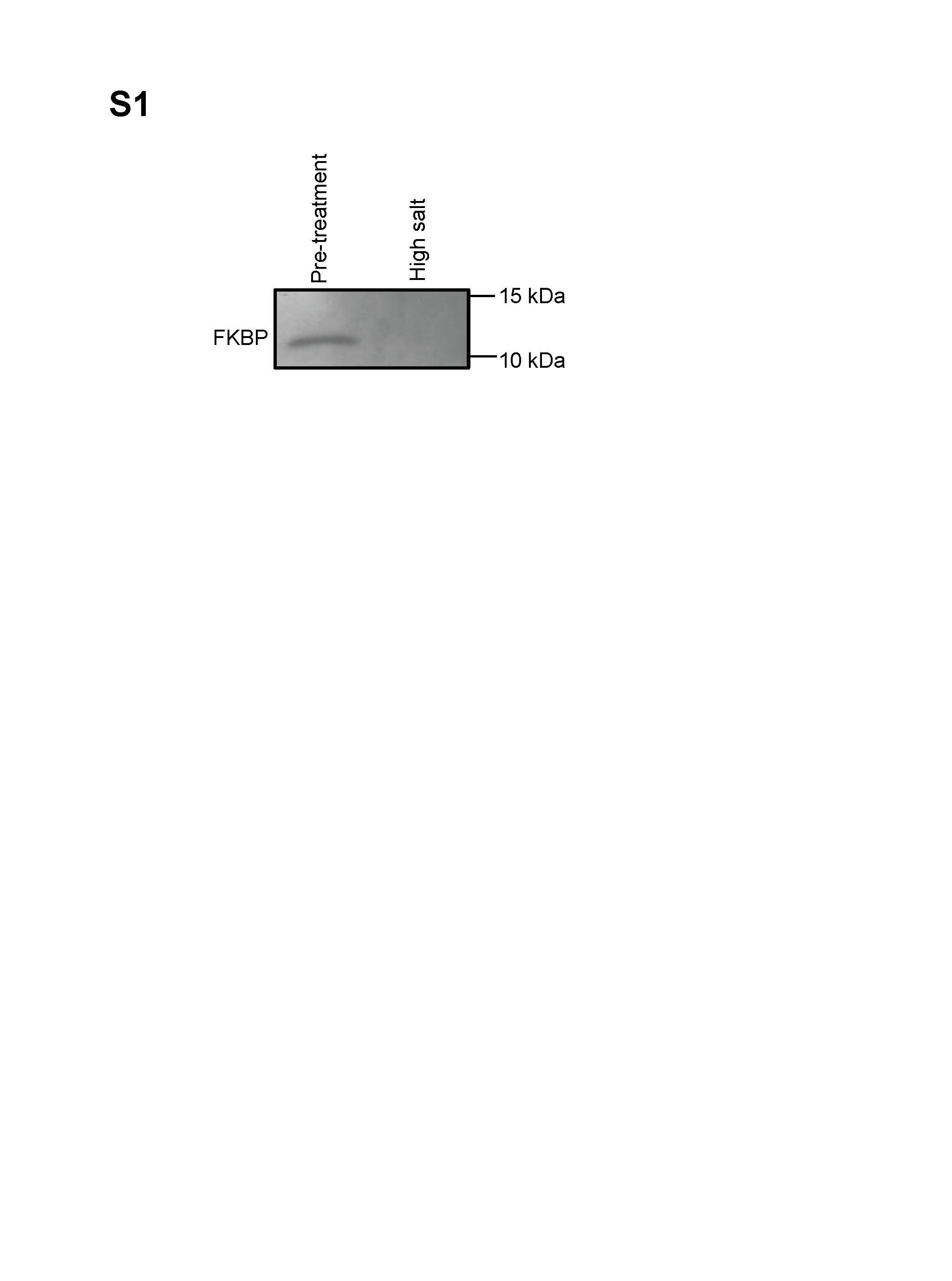

Supplement: Figure S1 — The effects of high salt incubation on the association of FKBPs with cardiac mixed membrane (MM) vesicles. Western blot analysis following incubation of cardiac mixed membrane vesicles with a high sucrose (0.4 M) solution (left lane) or a high salt (0.4 M) solution (right lane). Vesicles were loaded at 100 µg. Western blots were probed with an anti-FKBP12 antibody (recognising both FKBP12 and FKBP12.6). Size markers are indicated in kDa. (TIFF) [file pone.0031956.s001.tiff]
